# The Comparative Metabolism of a Novel Hepatocellular Carcinoma Therapeutic Agent, 2,3-Diamino-*N*-(4-(benzo[d]thiazol-2-yl)phenyl)propanamide, in Human and Animal Hepatocytes

**DOI:** 10.3390/metabo14080425

**Published:** 2024-08-01

**Authors:** Young-Heun Jung, Dong-Cheol Lee, Ye-Min Kwon, Eunbee Jang, Garam Choi, Yeoun-Hee Kim, Tae Hwan Kim, Ju-Hyun Kim

**Affiliations:** 1 College of Pharmacy, Yeungnam University, Gyeongsan 38541, Republic of Korea; jyoungheun@yu.ac.kr (Y.-H.J.); tbc99156@ynu.ac.kr (D.-C.L.); ym980822@yu.ac.kr (Y.-M.K.); 2 Etnova Therapeutics, Suwon 16648, Republic of Korea; wkddmsql96@etnova.co.kr (E.J.); garam1458@etnova.co.kr (G.C.); yhkim@etnova.co.kr (Y.-H.K.); 3 College of Pharmacy, Daegu Catholic University, Gyeongsan 38430, Republic of Korea

**Keywords:** ETN101, hepatocellular carcinoma, metabolism, hepatocyte, CYP, NAT

## Abstract

[2,3-diamino-*N*-(4-(benzo[d]thiazol-2-yl)phenyl)propanamide], named as ETN101, is a novel therapeutic agent for hepatocellular carcinoma. In vitro studies examined ETN101 metabolites in human, mouse, rat, dog, and monkey hepatocytes and identified the drug-metabolizing enzymes involved using cDNA-expressed human recombinant cytochrome P450s (CYPs), carboxylesterases (CESs), *N*-acetyltransferase (NAT) 1, and human liver cytosol. ETN101 showed similar metabolic stability across hepatocytes from five species, with particularly comparable stability in humans, rats, and monkeys. Its half-life was 75.0 min in humans, 68.9 in rats, 73.1 in monkeys, 120.4 in mice, and 112.7 in dogs. Thirty-four ETN101 metabolites, including the major metabolite M1, were identified using liquid chromatography–high-resolution mass spectrometry. ETN101 was primarily metabolized to M1 and CYP1A2 is exclusively responsible for M1 metabolism. Both NAT1 and NAT2 were responsible for the *N*-acetylation of M1 to M2. ETN101 remained stable in human CESs. In conclusion, this study provides comprehensive insights into the metabolic characteristics of ETN101, valuable for its toxicological and clinical development.

## 1. Introduction

Hepatocellular carcinoma (HCC) is one of the most prevalent liver cancers and a leading cause of cancer-related deaths globally [1]. Between 2005 and 2015, liver cancer became the second-largest contributor to cancer-related years of life lost worldwide, following lung cancer, with a 4.6% increase in absolute years of life lost (95% CI −1.6% to 15.4%) [2,3]. HCC often arises in the context of liver cirrhosis, characterized by liver scarring due to chronic liver disease [4]. Early-stage HCC is typically asymptomatic, complicating early diagnosis despite diagnostic advancements [5]. In addition, the aggressive nature of HCC, with its rapid growth and potential to metastasize, further complicates treatment and makes control difficult, particularly in advanced stages [6]. Chronic hepatitis B and C infections cause 80% of the HCC cases worldwide [2,7], with nonalcoholic fatty liver disease, diabetes mellitus, and alcoholic cirrhosis also being significant risk factors of HCC across countries [8,9,10,11].

Late diagnosis poses a significant challenge in HCC treatment. Surgical treatments, such as resection, transplantation, and ablation, require complex decisions based on tumor extent and liver function [12]. Multikinase inhibitors, such as sorafenib and lenvatinib, have been approved as first-line therapy for HCC [13,14,15]. However, resistance to sorafenib within six months of starting treatment and the lack of an approved second-line treatment after lenvatinib pose significant challenges [16]. Tyrosine kinase inhibitors such as regorafenib, cabozantinib, and ramucirumab have shown effectiveness as second-line therapeutics [17,18,19]. Recently, immune checkpoint inhibitors have emerged as promising therapeutic options for advanced-stage HCC [20]. The FDA approved nivolumab and pembrolizumab in 2017 and 2018, respectively, for patients previously treated with sorafenib [21,22]. However, as with other immune checkpoint inhibitors, adverse effects related to an imbalance in the immune system still require caution [23]. Despite the array of treatments, HCC patients typically experience a median progression-free survival of <1 year [24]. In addition, current therapies aim to extend survival but do not achieve complete remission, highlighting the need for the continued development of new therapeutic drugs to enhance HCC treatment strategies.

ETN101 [2,3-diamino-*N*-(4-(benzo[d]thiazol-2-yl)phenyl)propanamide] is currently in a phase 1 clinical development program in South Korea (ClinicalTrials.gov identifier: NCT06326502) as a novel HCC therapeutic. Its in vitro anticancer effects were demonstrated on liver cancer cell lines such as HepG2, Hep3B, Huh-7, and PLC/PRF/5. Additionally, the oral administration of ETN101 showed significant anticancer effects on subcutaneous xenograft tumors in nude mice and liver tumors in the HepG2 orthotopic animal model [25,26]. Based on these results, ETN101 is a promising anticancer agent for HCC and requires further evaluation through various drug development assays, including metabolism studies. In general, drug metabolism is a key process in regulating drug efficacy and toxicity after drug administration to a biological system [27]. However, undesirable metabolic characteristics may limit the development of the drug candidate which may be related to safety issues [28]. According to the typical chemical reactions, phase I metabolites tend to possess higher chemical reactivity or pharmacological activity, necessitating safety evaluations during drug development [29]. Therefore, drug metabolite characterization is essential, as the metabolites may contribute to either the beneficial aspect of therapy or serious toxicity, as highlighted in Safety Testing of Drug Metabolites Guidance for Industry [30] and the liquid chromatography coupled with mass spectrometric (LC–MS) detection is a common approach in the field due to its selectivity, accuracy, and versatility for targeted and untargeted analysis [31,32,33].

In this study, we comprehensively investigated the in vitro metabolic characteristics of ETN101 across the species, aiming to enhance our understating of ETN101 metabolism and ensuring its effective and safe utilization in clinical practice.

## 2. Materials and Methods

### 2.1. Chemicals and Reagents

ETN101 (alternative name: MBP-11901, batch no. MJY-MBP01-057, 99.5% purity) was provided from Etnova Therapeutics, which was produced by Hanmi Fine Chemical Co., Ltd. (Siheung-si, Republic of Korea). CJM-126 (2-(4-aminophenyl)benzothiazole, M1, batch no. 20201125-1, 99.1% purity) was obtained from Shanxi Chunjingcui Optoelectronics Technology Co., Ltd. (Xi’an, People’s Republic of China). The 6-OH-CJM-126 (M3, lot no. CS21058-BAJU0307, 99.7% purity) was purchased from Futurechem Co., Ltd. (Seoul, Republic of Korea), and the 3′-OH-CJM-126 (M5) and *N*-acetyl-M1 (M2) were provided by Etnova Therapeutics (Suwon, Republic of Korea) by in-house synthesis. NADPH regenerating system was purchased from Promega (Madison, WI, USA). Acetyl-coenzyme A sodium salt (≥93% purity), acetyl-DL-carnitine hydrochloride (>93% purity), phenacetin (99.8% purity), coumarin (≥99% purity), amodiaquine dihydrochloride dihydrate (100% purity), diclofenac sodium salt (100% purity), nifedipine (≥98.0% purity), oseltamivir phosphate (purity ≥ 98%), 1-aminobenzotriazole (99.0% purity), caffeic acid (≥98.0% purity), 4-aminosalicylic acid (99% purity) were purchased from Sigma (St. Louis, MO, USA). Carnitine acetyltransferase was purchased from Roche Diagnostics GmbH (Mannheim, Germany). Bupropion hydrobromide (100.0% purity) was purchased from The U.S. Pharmacopeial Convention, USP (Rockville, MD, USA). (S)-Mephenytoin (98% purity) and (S)-(+)-camptothecin (95% purity) were purchased from Toronto Research Chemicals (Toronto, ON, Canada). Bufuralol hydrochloride (99.5% purity) was purchased from Cayman Chemical (Ann Arbor, MI, USA). Chlorzoxazone (≥98.0% purity) was purchased from Tokyo Chemical Industry Co., Ltd. (Tokyo, Japan). Irinotecan (100.9% purity) was purchased from AVRA Laboratories PVT. Ltd. (Hyderabad, India). Cryopreserved human hepatocytes (mixed gender 50-donor pooled), INVITROGRO™ HT medium (Product No. Z99019), and INVITROGRO™ KHB (Product No. Z99074) were the products of BioIVT (Westbury, NY, USA). Corning Gentest mouse (male CD1, REF No. 454310), rat (male, REF No. 454701), dog (beagle, REF No. 454830, and monkey (cyno, REF No, 454930) cryopreserved hepatocytes; human recombinant cytochrome P450 (CYP) supersomes (2A6, 2C9, 2D6, 2E1, and 3A4); carboxylesterases (CES1b, CES1c, and CES2); and N-acetyltransferase (NAT) 1 were purchased from Corning Life Sciences (Woburn, MA, USA). Human liver cytosol fraction (Xtreme 200, H2610.C, Lot No. 1410229) was purchased from Sekisui Xenotech, LLC (Kansas City, MO, USA). Cryopreserved hepatocyte recovery medium for cryopreserved mouse, rat, dog, and monkey hepatocytes was obtained from Invitrogen (Cat No. CM7000, Waltham, MA, USA). Human recombinant CYP (1A2, 2B6, 2C8, and 2C19) were obtained from Cypex Ltd. (Dundee, UK). Acetonitrile and water (LC-MS grade) were obtained from Fisher Scientific Co. (Waltham, MA, USA).

### 2.2. Metabolic Stability

Cryopreserved hepatocytes were carefully thawed in a 37 °C water bath for approximately 1 min, and transferred to a tube containing pre-warmed thawing medium. The supernatants were removed and the pellets were resuspended in KHB at a final cell density of 1 × 10^6^ cells/mL, based on hemocytometer counting using trypan blue exclusion. A ETN101 solution was prepared by dissolving ETN101 in water at 4 mM and diluted by KHB at the concentration of 4 μM of ETN101. Sixty microliters each of hepatocyte suspensions and 4 μM of ETN101 were added in a 96-well plate and incubated for 0, 5, 15, 30, and 45 min at 37 °C in a CO_2_ incubator. All incubations were conducted in triplicates. Reactions were terminated by the addition of 120 μL of ice-cold acetonitrile and followed by vortex mixing. After centrifugation at 13,000 rpm for 8 min at 4 °C, 5 μL of internal standard (IS, camptothecin in acetonitrile at 780 ng/mL) was added to 60 μL of supernatant. A 2 μL aliquot was injected into the LC-MS/MS system. The reliability of the analysis was confirmed by incorporating three different levels of quality control samples at the beginning and end of each analytical batch. The disappearance rate (*k*) of ETN101 was calculated by determined peak area ratio of ETN101 to IS over incubation period. Subsequently, half-life (*t*_1/2_), intrinsic clearance (*Cl*_int_), and intrinsic hepatic clearance (*Cl*_int,hep_) of ETN101 in human and animal hepatocytes were calculated using the following equation [34]:

$$t_{1/2}(min)=\frac{ln2}{-k(elimination\;slope)}$$

$${Cl}_{int}\left(\mu L/min/10^{6}\;cells\right)=\frac{ln2}{t_{1/2}}\times \frac{\mu L\;incubation}{hepatocytes\;(10^{6}\;cells)}$$

$${Cl}_{int,hep}\left(mL/min/kg\right)=\frac{{Cl}_{int}}{1000}\times \frac{A\times 10^{6}\;cells}{g\;liver}\times \frac{B\;g\;liver}{kg\;body\;weight}$$

To calculate *Cl*_int,hep_, A has value of 139, 135, 117, 215, and 122; B has value of 25.7, 87.5, 40, 32, and 19.7, for human, mouse, rat, dog, and monkey, respectively.

### 2.3. Metabolite Characterization in Human and Animal Hepatocytes

We mixed 60 μL aliquots of 40 μM ETN101 in incubation medium and an equal volume of human, mouse, rat, dog, or monkey hepatocyte suspension in 96-well plates and incubated for 0 or 1 h in a CO_2_ incubator at 37 °C. The reactions were quenched by adding 120 µL of ice-cold acetonitrile to each well, and the cell suspension was sonicated for 5 min at 4 °C. Subsequently, centrifugation was carried out at 15,000× *g* for 10 min at 4 °C. Next, the supernatants were evaporated to dryness using a vacuum concentrator, and residues were re-dissolved in 70 μL of 30% acetonitrile. Each sample (5 μL aliquots) was analyzed using LC–HRMS system.

### 2.4. Screening of CYP Enzymes and CES Isozymes Responsible for the Metabolism of ETN101

ETN101 or M1 was incubated with nine human cDNA-expressed CYP enzymes (1A2, 2A6, 2B6, 2C8, 2C9, 2C19, 2D6, 2E1, and 3A4) to identify the CYPs involved in the metabolism of ETN101. Based on the preliminary study, the incubation conditions were determined as follows: Briefly, reaction mixtures of 50 μL containing ETN101 (final concentration: 10 μM) or M1 (final concentration: 1 μM), and CYPs (final amount: 5 pmol for CYP1A2, 2A6, 2C8, 2C9, 2C19, 2D6, and 3A4; 10 pmol for CYP2B6 and 2E1) were prepared in 0.1 M potassium phosphate buffer. The reaction was initiated by adding 50 μL of NADPH- regenerating system; the mixtures were incubated for 0 min or 30 min at 37 °C in triplicate. Reactions were terminated by the addition of 100 μL of ice-cold acetonitrile containing the IS (camptothecin, 200 ng/mL). Samples were centrifuged at 13,000 rpm for 5 min at 4 °C. An aliquot of 2 μL supernatant was injected in LC–MS/MS system.

To screen for CES enzymes responsible for the hydrolysis of ETN101 or M1, 50 μL of reaction mixtures containing human cDNA-expressed CES enzymes (CES1b, CES1c, and CES2) at the concentration of 1 mg/mL, 10 μM ETN101, or 1 μM M1 in 0.1 M potassium phosphate buffer were incubated at 37 °C for 0 min or 60 min in triplicate. The reactions were quenched by adding 50 μL of ice-cold acetonitrile containing the internal standard (camptothecin, 10 μg/mL). Following centrifugation at 13,000 rpm for 5 min, a 2 μL aliquot of the supernatant was injected into the LC-MS/MS system. The reliability of the analysis was confirmed by incorporating three different levels of quality control samples at the beginning and end of each analytical batch.

### 2.5. Characterization of Responsible NAT Isozymes for the Metabolism of ETN101

For the chemical inhibition assay, human liver cytosol was prepared in 0.05 M potassium phosphate buffer at a concentration of 1 mg/mL prior to use. A 1 μL of M1 (final concentration: 1 μM) solution was spiked in 223 μL of human liver cytosol preparations. One microliter each of caffeic acid and 1-aminobenzotriazole were added and incubated as NAT1 and NAT2 inhibitors, respectively [35,36]. The final concentrations of the NAT inhibitors were 0, 100, and 1000 μM in each incubate. After 5 min of pre-warming of the incubates at 37 °C, the metabolic reaction was initiated by adding 25 μL of acetyl-CoA regenerating system to the incubates. The samples were incubated in triplicate for 0 min or 30 min at 37 °C. The reaction was stopped by adding 250 μL of ice-cold acetonitrile containing the IS (camptothecin, 500 ng/mL) and vortex-mixing. Following centrifugation at 13,000 rpm for 5 min at 4 °C, a 2 μL aliquot of the supernatant was injected into the LC-MS/MS system. The percentage of remaining M1 and the formation of M2 were evaluated to determine the involvement of the NAT isozyme in *N*-acetylation of M1.

Additionally, M1 was incubated with cDNA-expressed human NAT1 isozyme to confirm the involvement of the NAT1 isozyme in the *N*-acetylation of M1. This was carried out by monitoring the remaining percentage of M1 and the formation of M2 in a concentration-dependent manner. A 225 μL reaction mixture containing cDNA-expressed human NAT1 at 1 μg/mL, and 1 μM M1 (final concentration: 0.1 and 1 μM) in 0.05 M potassium phosphate buffer was pre-warmed for 5 min. The reaction was initiated by adding 25 μL of acetyl-CoA regenerating system to the incubates. The acetyl-CoA regenerating system consisted of 0.1 mM acetyl-CoA, 4.5 mM acetyl-DL-carnitine, and 2.0 unit/mL carnitine acetyltransferase as final concentrations. The samples were incubated in triplicate for 0 min or 60 min at 37 °C. The reactions were stopped by adding 250 μL of ice-cold acetonitrile containing the IS (camptothecin, 500 ng/mL). Samples were centrifuged at 13,000 rpm for 5 min at 4 °C. An aliquot of 2 μL supernatant was injected in LC-MS/MS system.

### 2.6. LC-MS Analysis

For the metabolic stability assay, an LC-MS/MS system was employed, consisting of an Agilent 1260 series HPLC system (Agilent Technologies Inc., Santa Clara, CA, USA) coupled to an API 4000 mass spectrometer (AB SCIEX, Concord, Vaughan, ON, Canada) equipped with a turbo electrospray interface. The analyte and IS were separated and eluted from the Kinetex Halo^®^ C18 column (2.7 μm, 2.1 × 50 mm, Advanced Material Technology, Wilmington, DE, USA) using a gradient elution of 0.1% (*v*/*v*) formic acid in water (mobile phase A) and 0.1% (*v*/*v*) formic acid in acetonitrile (mobile phase B) at the column temperature of 40 °C. The gradient elution program proceeded as follows: starting with 10% B for 0.5 min, followed by a linear increase to 80% B over 0.2 min, maintaining 80% B for 2.5 min, and then returning to initial conditions over 0.2 min. The column was subsequently reconditioned using 10% B for 4.6 min. The flow rate was 0.3 mL/min. ETN101 and IS were detected using multiple reaction monitoring (MRM) mode under positive electrospray ionization (ESI). The ESI source conditions for the ionizations of the target compounds were as follows: gas temperature, 450 °C; collision gas, 6 psi; curtain gas, 20 psi; ion source gas 1, 40 psi; ion source gas 2, 50 psi; and ion spray voltage, 5.5 kV. The collision energy voltages were optimized to 25 and 33 V for ETN101 and IS, respectively. The declustering potentials were optimized to 66 V for ETN101 and 75 V for the IS. The optimized collision cell exit potentials were at 18 V for ETN101 and 15 V for the IS. The MRM transitions of *m*/*z* 313.2 ⟶ 227.0 and *m*/*z* 349.2 ⟶ 305.1 were selected to detect ETN101 and IS, respectively. Data acquisition and processing were performed using the Analyst software (version 1.5.2, Sciex, Framingham, MA, USA).

For metabolite characterization, a Vanquish HPLC system (Thermo Fisher Scientific, San José, CA, USA) coupled with a Q-Exactive Orbitrap mass spectrometer (Thermo Fisher Scientific Inc., Waltham, MA, USA) was used at Core Research Support Center for Natural Products and Medical Materials at Yeungnam University. Analytes were separated in a HALO^®^ C18 column (2.7 μm, 2.1 × 100 mm, Advanced Material Technology, Wilmington, DE, USA) using a gradient elution of 0.1% (*v*/*v*) formic acid in water (mobile phase A) and 0.1% (*v*/*v*) formic acid in acetonitrile (mobile phase B) at the column temperature of 40 °C. The gradient elution program involved starting with 10% B for 3 min, gradually increasing to 80% B over 9 min, maintaining 80% B for 1.5 min, then returning to initial conditions over 0.1 min, followed by column reconditioning using 10% B for 1.4 min at a flow rate of 0.3 mL/min. The eluent from the column was directly injected into the mass spectrometer using a heated electrospray ionization (HESI) source in positive ionization mode. The sheath and auxiliary gas flow rates were 45 and 11.25 (arbitrary units), respectively. The spray voltage was 3.0 kV and the capillary temperature was 320 °C. Full scan MS^1^ with a data-dependent MS^2^ acquisition mode was applied to obtain MS^1^ scan data ranging from *m*/*z* 80 to 900, at a resolution of 70,000, whereas data-dependent MS^2^ spectra were acquired for the five most abundant peaks per cycle at a resolution of 35,000. The parallel reaction monitoring (PRM) mode was used to obtain the MS^2^ spectra of putative metabolites detected at low peak intensities in the data-dependent MS^2^ acquisition mode. Data were collected and interpreted using Thermo Scientific Xcalibur™ Software (Thermo Fisher Scientific).

For enzyme characterization, an Agilent 6460 triple quadrupole mass spectrometer coupled with a 1260 HPLC system (Agilent Technologies, Santa Clara, CA, USA) and a Kinetex Halo^®^ C18 column (2.7 μm, 2.1 × 50 mm, Advanced Material Technology, Wilmington, DE, USA) were used for chromatographic separation. The mobile phases comprised 0.1% (*v*/*v*) formic acid in water (mobile phase A) and 0.1% formic acid (*v*/*v*) in acetonitrile (mobile phase B). The mobile phase flowed at a rate of 0.3 mL/min, and the gradient elution program proceeded as follows: starting with 10% B for 0.5 min, a linear increase to 90% B for 0.2 min, maintaining 90% B for 1.5 min, and then returning to initial conditions for 0.2 min. Subsequently, the column was reconditioned using 10% B for 2.6 min. The column and auto-sampler were maintained at 40 °C and 4 °C, respectively. ETN101, M1, and M2 were detected via MRM using ESI in the positive mode. The ion source conditions were optimized as follows: gas temperature: 330 °C; gas flow: 5 L/min; nebulizer: 45 psi; sheath gas temperature: 250 °C; sheath gas flow: 11 L/min; capillary voltage: 3500 V; nozzle voltage: 500 V. The MRM transitions for ETN101 were *m*/*z* 313.2 ⟶ 227.0 at a collision energy (CE) of 14. The MRM transitions for M1 and M2 were *m*/*z* 227.2 ⟶ 109.0 at a CE of 30 and *m*/*z* 269.1 ⟶ 227.1 at a CE of 26, respectively. For IS (camptothecin), the MRM transition of *m*/*z* 349.2 ⟶ 305.1 at a CE of 33 was used. Mass Hunter software (Agilent Technologies) was used to control the LC-MS/MS instrument and for data processing.

## 3. Results

### 3.1. Metabolic Stability of ETN101 in Hepatocytes

**Figure 1:**
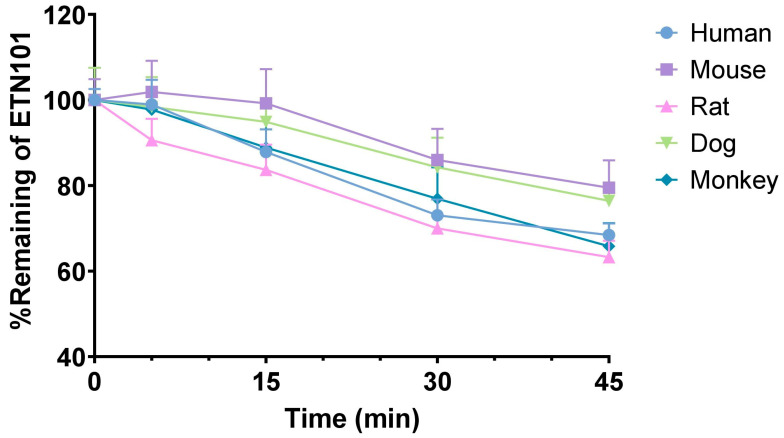
Percentage of ETN101 remaining after incubation with human, mouse, rat, dog, and monkey hepatocytes at 37 °C in a CO_2_ incubator. The symbol and error bar represent mean and standard deviation, respectively (n = 3, except human, where n = 4).

The percentage of ETN101 remaining after incubation with human, mouse, rat, dog, and monkey hepatocytes is presented in Figure 1 and the in vitro metabolic stability parameters of ETN101 in human, mouse, rat, dog, and monkey hepatocytes are shown in Table 1. The *t*_1/2_ values of ETN101 after 45 min of incubation in human, mouse, rat, dog, and monkey hepatocytes were 75.0, 120.4, 68.9, 112.7, and 73.1 min, respectively. *Cl*_int_ were 18.5, 11.5, 20.1, 12.3, and 19.0 μL/min/10^6^ cells, respectively. Based on these findings, the metabolic stability of ETN101 was similar among the human, rat, and monkey hepatocytes, whereas its stability in the mouse and dog hepatocytes was higher than that of the other three species.

### 3.2. Metabolite Characterization of ETN101 in Hepatocytes

**Figure 2:**
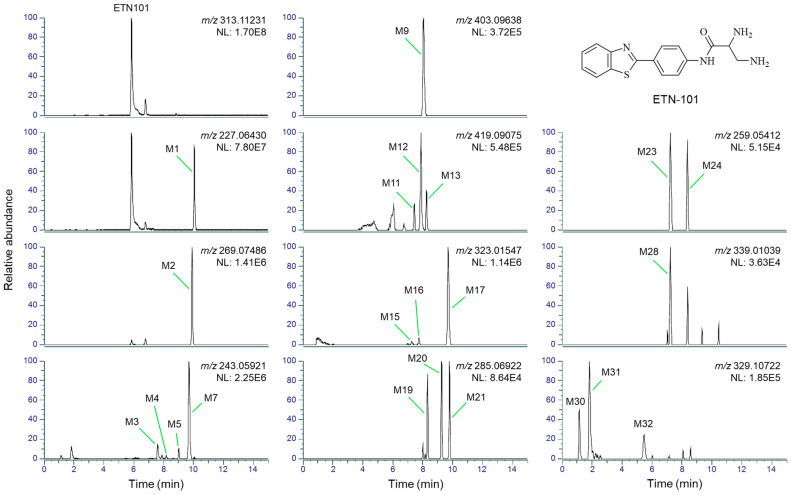
The chemical structure of ETN101 and representative extracted ion chromatograms of ETN101 and its metabolites identified in human hepatocyte incubates (NL: normalized level).

ETN101 was metabolized to 16 phase I metabolites (M1, M3–M8, M23–M26, M30–M34) and 18 phase II metabolites (M2, M9–M22, M27–M29) after incubation with human, mouse, rat, dog, or monkey hepatocytes. The chemical structure of ETN101 and the representative extracted ion chromatograms (EICs) of ETN101 and its metabolites in human hepatocytes are shown in Figure 2. The EICs of ETN101 and its metabolites observed in mouse, rat, dog, and monkey hepatocytes are presented in the Supplementary Materials (Figures S1–S4). The molecular formulae, exact mass values, retention times, product ions, and biotransformation pathways of ETN101 and its metabolites are summarized in Table 2.

**Figure 3:**
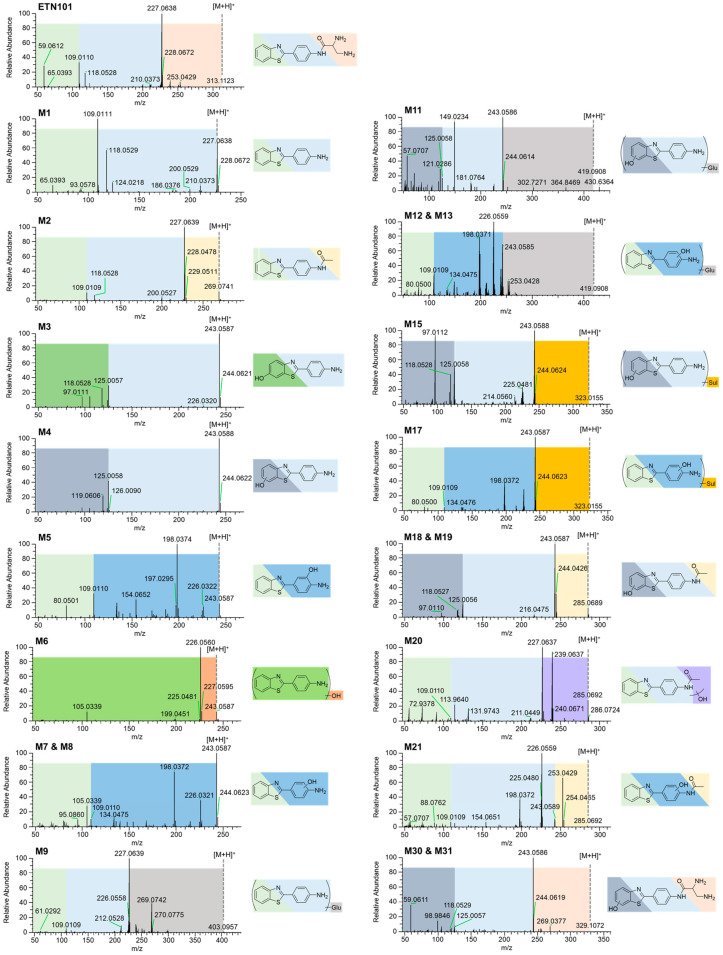
MS/MS spectra of ETN101 and its metabolites identified in human and animal hepatocyte incubates. Identical fragment ions are depicted in the same color.

ETN101 was observed at a retention time (t_R_) of 5.9 min and exhibited an [M + H]^+^ ion at *m*/*z* 313.1123. The characteristic product ions were *m*/*z* 65.0393, *m*/*z* 109.0010, *m*/*z* 118.0528, and *m*/*z* 227.0638 according to the MS/MS spectrum. These product ions serve as fragment ion markers for metabolite structural characterization (Figure 3).

M1 was observed at t_R_ 10.1 min and exhibited an [M + H]^+^ ion at *m*/*z* 227.0643, indicating the formation of an amide bond-hydrolyzing metabolite. M1 produced product ions at *m*/*z* 65.0393, *m*/*z* 109.0111, and *m*/*z* 118.0529. M1 was identified as CJM-126 by comparing t_R_ with that of an authentic standard.

M2 was observed with a t_R_ of 9.9 min, exhibiting an [M + H]^+^ ion at *m*/*z* 269.0749, which is 42.0106 atomic mass units (amu) higher than the [M + H]^+^ ion of M1, suggesting its formation through the *N*-acetylation of M1. M2 produced product ions at *m*/*z* 65.0.91, *m*/*z* 109.0109, *m*/*z* 118.0528, and *m*/*z* 227.0639. M2 was identified as *N*-acetyl-CJM-126 by comparing t_R_ with that of an authentic standard.

M3, M4, M5, M6, M7, and M8 were observed at a t_R_ of 7.6, 8.2, 9.0, 9.5, 9.7, and 10.1 min, respectively, and exhibited an [M + H]^+^ ion at *m*/*z* 243.0592, which is 15.9949 amu higher than the [M + H]^+^ ion of M1, indicating that they were formed by monohydroxylation. M3 yielded product ions at *m*/*z* 81.0341, *m*/*z* 118.0528, and *m*/*z* 125.0057. M3 was identified as 6-hydroxy-CJM-126 by comparing the t_R_ of M3 with the authentic standard. M4 yielded product ions at *m*/*z* 81.0340 and *m*/*z* 125.0058, indicating that hydroxylation site was on the benzene ring of the 1,3-benzothiazole moiety. M5 produced product ions at *m*/*z* 65.0393, *m*/*z* 109.0110, and *m*/*z* 134.0476. M5 was identified as 3′-hydroxy-CJM-126 by comparing the t_R_ of M5 with the authentic standard. The exact hydroxylation position of M6 could not be able to be confirmed due to the lack of product ion. M7 and M8 yielded product ions at *m*/*z* 109.0110 and *m*/*z* 134.0475, indicating the hydroxylation of the benzene ring of the aniline moiety.

M9, observed at a t_R_ of 8.1 min, exhibited an [M + H]^+^ ion at *m*/*z* 403.0964, which is 176.0321 amu higher than the [M + H]^+^ ion of M1, suggesting its formation through the glucuronidation of M1. M9 produced product ions at *m*/*z* 109.0109, *m*/*z* 118.0527, and *m*/*z* 227.0639.

M10 was observed at a t_R_ of 9.1 min and exhibited an [M + H]+ ion at *m*/*z* 307.0211, which is 79.957 amu greater than the [M + H]^+^ ion of M1, indicating that it was formed by sulfation on M1. MS/MS spectra were not acquired because of their low abundance in all hepatocytes incubated throughout the species.

M11 was observed at a t_R_ of 7.4 min and exhibited an [M + H]^+^ ion at *m*/*z* 419.0908, which is 176.0316 amu greater than the [M + H]^+^ ion of the mono-hydroxylated M1, indicating that it was formed by glucuronidation on the hydroxylated M1. M11 produced product ions at *m*/*z* 118.0529, *m*/*z* 125.0058, and *m*/*z* 243.0586, indicating that glucuronide was conjugated to either M3 or M4.

M12 and M13 were observed at a t_R_ of 7.9 min and 8.3 min, respectively, and exhibited an [M + H]^+^ ion at *m*/*z* 419.0908, which is 176.0316 amu greater than the [M + H]^+^ ion of the mono-hydroxylated M1, suggesting their formation through glucuronidation of the hydroxylated M1. M12 and M13 produced product ions at *m*/*z* 65.0392, *m*/*z* 109.0109, *m*/*z* 134.0475, and *m*/*z* 243.0585, indicating that the glucuronide was conjugated to M5, M7, or M8.

M14, M15, M16 and M17 were observed at a t_R_ of 7.0, 7.3, 7.8, and 9.7 min, respectively, and exhibited an [M + H]^+^ ion at *m*/*z* 323.0155, which is 79.9563 amu greater than the [M + H]^+^ ion of the mono-hydroxylated M1, indicating that it was formed by sulfation on the hydroxylated M1. M15 yielded product ions at *m*/*z* 118.0528, *m*/*z* 125.0058, and *m*/*z* 243.0588, indicating that the sulfate was conjugated to M3 or M4. M17 yielded product ions at *m*/*z* 109.0109, *m*/*z* 134.0476, *m*/*z* 243.0587, indicating that sulfate was conjugated to M5, M7, or M8.

M18, M19, M20, and M21 were observed at a t_R_ of 8.0, 8.3, 9.3, and 9.8 min, respectively, and exhibited an [M + H]^+^ ion at *m*/*z* 285.0692, which is 42.0100 amu greater than the [M + H]^+^ ion of the mono-hydroxylated M1, indicating that it was formed by acetylation on the hydroxylated M1. M18 and M19 yielded product ions at *m*/*z* 81.0341, *m*/*z* 118.0527, *m*/*z* 125.0056, and *m*/*z* 243.0587, indicating the *N*-acetylation of the aniline moiety of either M3 or M4. M20 yielded product ions at *m*/*z* 109.0110 and *m*/*z* 227.0637, indicating that the hydroxylation of the acetylated moiety of M2. M21 yielded product ions at *m*/*z* 109.0109 and 243.0589, indicating the hydroxylation on the benzene ring of the acetanilide moiety.

M22 was observed at a t_R_ of 6.4 min, and exhibited an [M + H]^+^ ion at *m*/*z* 461.1013, which was 176.0321 amu greater than the [M + H]^+^ ion of mono-hydroxylated M2, indicating the glucuronidation of M18, M19, M20, or M21. The exact position of the glucuronidation could not be confirmed because of the absence of MS/MS spectra.

M23, M24, M25, and M26 were observed at a t_R_ of 7.2 min, 8.4 min, 9.5 min and 10.4 min, respectively, and exhibited an [M + H]^+^ ion at *m*/*z* 259.0541, which is 31.9898 amu greater than the [M + H]^+^ ion of M1, indicating a dihydroxylation on M1. The exact position of dihydroxylation could not be confirmed because of the absence of MS/MS spectra.

M27, M28, and M29 observed at a t_R_ of 7.0 min, 7.2 min, and 8.4 min, respectively, and exhibited the [M + H]^+^ ion at *m*/*z* 339.0104, which was 79.9563 amu greater than the [M + H]^+^ ion of dihydroxylated M1, indicating the sulfation of M23, M24, M25, or M26. The exact position of dihydroxylation could not be confirmed because of the absence of MS/MS spectra.

M30, M31, M32, M33, and M34 was observed at a t_R_ of 1.1 min, 1.8 min, 5.5 min, 7.1 min, and 7.9 min, respectively, and exhibited an [M + H]^+^ ion at *m*/*z* 329.1072, which is 15.9949 amu greater than the [M + H]^+^ ion of ETN101, indicating a mono-hydroxylation on ETN101. M30 and M31 yielded product ions at *m*/*z* 118.0529, *m*/*z* 125.0057, and *m*/*z* 243.0586 indicating that hydroxylation site was on the benzene ring of the 1,3-benzothiazole moiety. The exact position of hydroxylation could not be confirmed for M32, M33, and M34 because of the absence of MS/MS spectra.

### 3.3. Characterization of Drug-Metabolizing Enzymes Responsible for ETN101 Metabolism

#### 3.3.1. Human cDNA-Expressed CYP Enzymes

**Figure 4:**
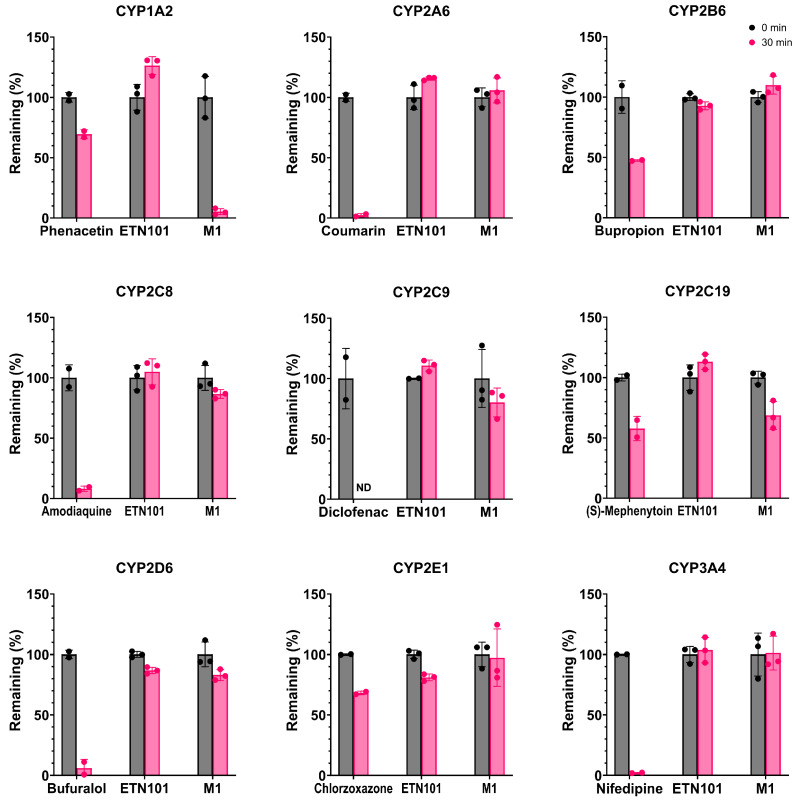
Percentage remaining of ETN101 and M1 after incubation with nine human cytochrome P450s (CYPs) at 37 °C for 30 min. To demonstrate the activity of each enzyme, probe substrates were used as a positive control for each CYP (phenacetin for 1A2; coumarin for 2A6; bupropion for 2B6; amodiaquine for 2C8; diclofenac for 2C9; (S)-mephenytoin for 2C19; bufuralol for 2D6; chlorzoxazone for 2E1; and nifedipine for 3A4) [37,38,39]. The error bar represents standard deviation (n = 2 for positive control or n = 3 for ETN101 or M1). ND indicates not detected.

To characterize the CYP enzymes responsible for the metabolism of ETN101 or M1, we evaluated the percentage of ETN101 or M1 remaining after 30 min of incubation with nine human cDNA-expressed CYP enzymes in the presence of NADPH. ETN101 levels remained at 126.3, 115.6, 92.8, 105.0, 110.8, 113.0, 86.9, 81.1, and 103.5% after 30 min of incubation with CYP1A2, 2A6, 2B6, 2C8, 2C9, 2C19, 2D6, 2E1, and 3A4, respectively, suggesting a small contribution of CYPs to the metabolism of ETN101 (Figure 4). In contrast, M1 remained at 5.3, 105.9, 109.8, 86.6, 80.1, 68.7, 83.0, 97.3, and 101.1% after 30 min of incubation with CYP1A2, 2A6, 2B6, 2C8, 2C9, 2C19, 2D6, 2E1, and 3A4, respectively, indicating that CYP1A2 predominantly mediates the metabolism of M1, with a minor contribution from CYP2C19 (Figure 4).

#### 3.3.2. Human Recombinant CES Isozymes

**Figure 5:**
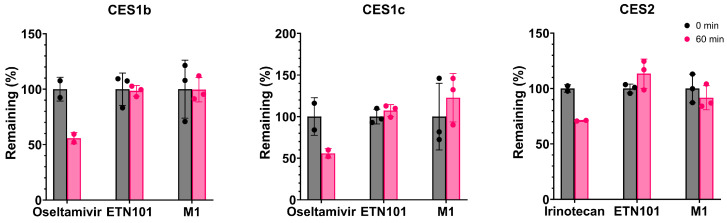
Percentage remaining of ETN101 and M1 after incubation with three human carboxylesterases (CESs) at 37 °C for 60 min. To demonstrate the activity of each enzyme, probe substrates were used as a positive control for each CES (oseltamivir for CES1b and CES1c; irinotecan for CES2) [40,41]. The error bar represents standard deviation (n = 2 for positive control or n = 3 for ETN101 or M1).

To characterize the CES isozymes involved in the metabolism of ETN101 or M1, the percentage of ETN101 or M1 remaining after 60 min of incubation with three human recombinant CES isozymes was determined. After 60 min of incubation with CES1b, CES1c, and CES2, ETN101 remained at 98.6%, 107.3%, and 113.4%, respectively (Figure 5). These results suggest that CESs are not involved in the metabolism of ETN101. Similarly, M1 remained at 99.6, 122.7, and 91.7%, indicating that CESs do not mediate the metabolism of M1 (Figure 5).

#### 3.3.3. Human Liver Cytosolic NAT1 and NAT2

**Figure 6:**
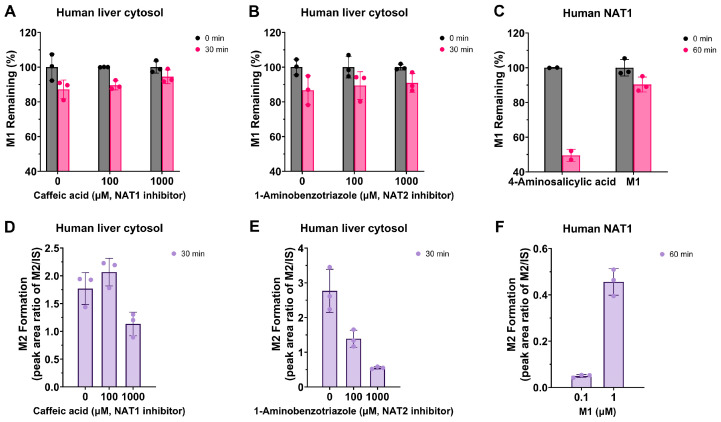
Percentage remaining of M1 after incubation in human liver cytosol with or without *N*-acetyltransferases (NATs) inhibitor, caffeic acid for NAT1 (**A**) or 1-aminobenzotriazole for NAT2 (**B**), at 37 °C for 30 min. Percentage remaining of M1 after incubation in human NAT1 at 37 °C for 60 min (**C**). Peak area ratio of M2 after incubation of M1 in human liver cytosol with or without caffeic acid (**D**) or 1-aminobenzotriazole (**E**) at 37 °C for 30 min. Peak area ratio of M2 after the incubation of M1 (0.1 and 1 μM) in cDNA-expressed human NAT1 at 37 °C for 60 min (**F**). The 4-aminosalicylic acid served as a positive control for metabolism mediated by NAT1 [42]. The error bar represents standard deviation (n = 2 for positive control or n = 3 for M1).

To investigate the involvement of NAT1 or NAT2 in the metabolism of M1, the percentages of M1 remaining after incubation with human liver cytosol in the presence of caffeic acid or 1-aminobenzotriazole, which served as selective inhibitors for NAT1 and NAT2, respectively, were evaluated. After 30 min of incubation in human liver cytosol with caffeic acid at concentrations of 0, 100, and 1000 μM, M1 remained at 87.2%, 89.6%, and 94.4%, respectively (Figure 6A). With 1-aminobenzotriazole at concentrations of 0, 100, and 1000 μM, M1 remained at 86.7%, 89.4%, and 90.9%, respectively (Figure 6B).

Although the trend in remaining percentage increases with the concentration of inhibitor, these results were insufficient to conclude the involvement of NAT1 or NAT2 in the *N*-acetylation of M1 to M2. Therefore, the peak area ratio of M2 was monitored to confirm the role of NATs in the metabolism of M1 to M2. Finally, the observed decrease in the peak area ratio of M2 with increasing inhibitor concentration indicated the involvement of NAT1 and NAT2 in the *N*-acetylation of M1 to M2 (Figure 6D,E).

#### 3.3.4. Human Recombinant NAT1

The percentages of M1 remaining after incubation with cDNA-expressed human NAT1 was determined. M1 remained at 90.3% after 60 min of incubation with NAT1 at a concentration of 0.1 μM, tentatively confirming the involvement of NAT1 in the *N*-acetylation of M1 (Figure 6C). To further verify the role of NAT1 in the *N*-acetylation of M1, the peak area ratio of M2 was monitored. The M2 signal increased proportionally with the concentration of M1 after 60 min of incubation with NAT1 (Figure 6F). Taken together, these results indicate that NAT1 is responsible for the *N*-acetylation of M1 to M2.

## 4. Discussion

The metabolic stability of ETN101 in human, mouse, rat, dog, and monkey hepatocytes resulted in the intrinsic clearance of 18.5, 11.5, 20.1, 12.3, and 19.0 μL/min/10^6^ cells, respectively. These findings indicate that the stability of ETN101 is similar across humans, rats, and monkeys compared to that in mice and dogs. Assessing ETN101’s metabolic stability in hepatocytes across species is essential for predicting human pharmacokinetics from animal studies and interpreting toxicity and pharmacokinetic evaluations [43]. Considering the intrinsic clearance and half-life of ETN101, rats and monkeys showed similar patterns to humans compared to the other two species tested.

**Figure 7:**
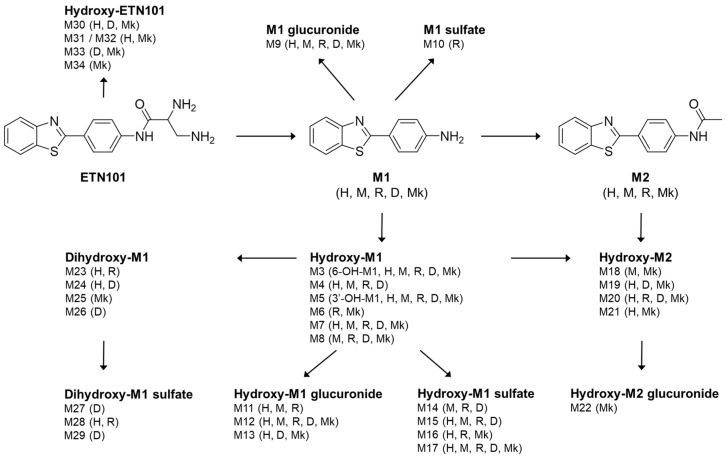
Proposed metabolic pathways of ETN101 in hepatocyte incubates (H: human, M: mouse, R: rat, D: dog, Mk: monkey).

A total of 34 metabolites of ETN101 were characterized in hepatocyte incubates across species. These include CJM-126 (M1, CAS No. 6278-73-5), *N*-acetyl-M1 (M2), hydroxy-M1 (M3–M8), M1 glucuronide (M9), M1 sulfate (M10), hydroxy-M1 glucuronide (M11–M13), hydroxy-M1 sulfate (M14–M17), hydroxy-M2 (M18–M21), hydroxy-M2 glucuronide (M22), dihydroxy-M1 (M23–M26), dihydroxy-M1 sulfate (M27–M29), and hydroxy-ETN101 (M30–M34) (Figure 7). The relative amounts of each metabolite are shown in Supplementary Table S1.

The most commonly observed metabolite of ETN101 was M1, which was formed by the hydrolysis of carboxamide on ETN101. Six hydroxy-M1 (M3–M8) were detected at different retention times, indicating hydroxylation at different sites on M1. M1, M3 (6-OH-CJM-126), and M5 (3′-OH-CJM-126) were identified using authentic standards. M9 and M10 are glucuronide- and sulfate-conjugated M1, respectively. Three hydroxy-M1 glucuronides (M11–M13) were characterized based on their accurate molecular ion masses and MS/MS spectra. The representative product ions of M11 suggested that glucuronide was conjugated to either M3 or M4, whereas glucuronide was conjugated on either M5, M7, or M8 for M12 and M13. The exact site of the glucuronidation was unable to be confirmed, since only the neutral loss of glucuronide (−176 Da) is commonly observed in glucuronide-conjugated metabolites. Similarly, four hydroxy-M1 sulfates (M14–M17) were characterized, and putative structures of M14 and M16 were suggested based on their MS/MS spectra. Since five glucuronide-conjugated metabolites (M9, M11-M13, M22) and eight sulfate-conjugated metabolites (M10, M14-M17, M27-M29) were identified in the hepatocyte incubates across the species, it is recommended that the enzymes responsible for the phase II metabolism of ETN101 are explored when necessary.

M2 was the major metabolite after M1, except in dogs, and was identified by comparing the retention time and MS/MS spectra with those of the authentic standard. Four hydroxy-M2 (M18–M21) metabolites were observed at separate retention times, and putative structures were suggested based on their respective MS/MS spectra. Dihydroxy-M1 (M23–M26) and dihydroxy-M1 sulfate (M27–M29) were minor metabolites with low signal intensities across species, which limited the acquisition of MS/MS spectra.

ETN101 or M1 were incubated with cDNA-expressed human CYPs and CESs to identify the drug-metabolizing enzymes responsible for ETN101 or M1 metabolism. ETN101 was relatively stable across the nine CYPs, whereas CYP1A2 was predominantly involved in M1 metabolism compared to the other enzymes. Monitoring the M1 signal after incubating ETN101 with CYPs indicated the weak involvement of CYP2E1. Since the percentage of remaining ETN101 was insufficient to determine the role of CYP2E1 in ETN101 metabolism, further investigation into CYP2E1’s involvement, such as examining its intrinsic clearance, may help clarify the role of CYP2E1 in ETN101 metabolism. Both ETN101 and M1 were metabolically stable in the recombinant CESs tested. CESs play a critical role in drug metabolism and pharmacokinetics by catalyzing the hydrolysis of esters, amides, carbamates, and thioesters [44]. Considering the chemical structure of ETN101, the carboxamide moiety of ETN101 was expected to be hydrolyzed by CESs but was found to be stable in CES1b, CES1c and CES2. These findings suggest that the principal enzyme responsible for ETN101 hydrolysis requires further investigation and if so, the kinetic of ETN101 metabolism to M1 needs to be evaluated.

As six *N*-acetylation-related metabolites (M2, M18–M22) were characterized, *N*-acetyltransferases were expected to be involved in ETN101 metabolism. Both NAT1 and NAT2 were found to be involved in the *N*-acetylation of M1, based on the remaining (%) of M1 after incubation with cDNA-expressed human NAT1 or human liver cytosol, with or without NAT inhibitors. Additionally, by monitoring M2 formation in each incubate, we confirmed the involvement of NAT1 and NAT2 in the *N*-acetylation of M1 to M2.

Although the anti-tumor effects of ETN101 have been confirmed in previous studies, its major metabolite, M1 [2-(4-aminophenyl) benzothiazole, CJM-126], also shows significant anti-cancer properties [45]. The National Cancer Institute in the USA identified CJM-126 derivatives as a unique class of potent aryl hydrocarbon receptor (AhR) agonists [46]. Binding to AhR induces CYP1A1, which produces reactive species that form DNA adducts [47,48,49]. Ultimately, this process results in cell death through the activation of apoptotic machinery. Particularly, CYP1A1 is known to be involved in the benzothiazole metabolism, and the CYP1A2-mediated significant depletion of M1 is not surprising regarding the high similarity of CYP1A family [46,50]. In this study, we evaluated CYP1A2, as it plays major role in drug metabolism; the involvement of CYP1A1 in the metabolism of ETN101 or M1 is highly encouraged in the future. In vitro studies have confirmed that the major CJM-126 metabolic pathways, which were *N*-oxidation, C-6 oxidation, and *N*-acetylation, depended on the derivative structures [46,51]. *N*-acetyl-CJM-126 and 6-hydroxy-CJM-126, identified in this study as M2 and M3, respectively, have been confirmed by commercially available chemicals or in-house synthesized standards, and the *N*-acetylation of CJM-126 was previously confirmed as a major metabolite of CJM-126 through an in vitro study [51].

In summary, we identified a total of 34 metabolites of ETN101 from human and animal hepatocyte incubations, including two known metabolites, M1 (CJM-126) and M2 (*N*-acetyl-CJM-126). While ETN101 is currently developed as a racemate, chiral isomers can exhibit different metabolic characteristics; therefore, the metabolic characteristics of ETN101 isomers should be investigated when they are available. Additionally, although this study mainly focused on the hepatocyte-level metabolism of ETN101, incorporating cellular fraction-level metabolism, such as liver microsomes and S9 fractions, can enhance our understanding of metabolic characteristics, particularly reaction phenotypes.

## 5. Conclusions

ETN101, a novel agent for HCC treatment, was metabolized to 34 metabolites in human, mouse, rat, dog, and monkey hepatocytes. ETN101 was primarily metabolized to M1 and CYP1A2 was predominantly involved in M1 metabolism compared to other CYPs. NAT1 and NAT2 are responsible for the *N*-acetylation of M1 to M2. Although ETN101 is stable in human CYPs and CESs, its major metabolite, M1, undergoes extensive metabolism, and there is still a need to identify the primary metabolic enzymes involved in ETN101 metabolism beyond those evaluated in this study.

## Figures and Tables

**Table 1:** Half-life, intrinsic clearance, and intrinsic hepatic clearance values of ETN101 in human, mouse, rat, dog, and monkey hepatocytes after 45 min incubation at 37 °C.

Parameters	Human	Mouse	Rat	Dog	Monkey
t_1/2_ (min)	75.0	120.4	68.9	112.7	73.1
*Cl*_int_ (μL/min/10^6^ cells)	18.5	11.5	20.1	12.3	19.0
*Cl*_int,hep_ (mL/min/kg)	66.1	135.8	94.1	84.6	45.7

**Table 2:** Molecular formulae, exact mass, retention times (t_R_), and product ions of ETN101 and its metabolites identified in human, mouse, rat, dog, and monkey hepatocytes (H: human; M: mouse; R: rat; D: dog; Mk: monkey).

ID	ETN101 and Metabolites	Elemental Composition	Exact Mass ([M + H]^+^)	t_R_ (min)	Product Ions (*m*/*z*)	Species
	ETN101	C_16_H_16_N_4_OS	313.1123	5.9	65.0393, 109.0110, 118.0528, 227.0638	
**Hydrolysis**						
M1	CJM-126	C_13_H_10_N_2_S	227.0643	10.1	65.0393, 109.0111, 118.0529	H, M, R, D, Mk
***N*-acetylation of M1**						
M2	*N*-Acetyl-CJM-126	C_15_H_12_N_2_OS	269.0749	9.9	65.0391, 109.0109, 118.0528, 227.0639	H, M, R, Mk
**Hydroxylation of M1**						
M3	6-OH-CJM-126	C_13_H_10_N_2_OS	243.0592	7.6	81.0341, 118.0528, 125.0057	H, M, R, D, Mk
M4	OH-CJM-126	C_13_H_10_N_2_OS	243.0592	8.2	81.0340, 125.0058	H, M, R, D
M5	3′-OH-CJM-126	C_13_H_10_N_2_OS	243.0592	9.0	65.0393, 109.0110, 134.0476	H, M, R, D, Mk
M6	OH-CJM-126	C_13_H_10_N_2_OS	243.0592	9.5	Not acquired	R, Mk
M7	OH-CJM-126	C_13_H_10_N_2_OS	243.0592	9.7	109.0110, 134.0475	H, M, R, D, Mk
M8	OH-CJM-126	C_13_H_10_N_2_OS	243.0592	10.1	109.0110, 134.0475	M, R, D, Mk
**Glucuronidation of M1**						
M9	CJM-126 glucuronide	C_19_H_18_N_2_O_6_S	403.0964	8.1	109.0109, 118.0527, 227.0639	H, M, R, D, Mk
**Sulfation of M1**						
M10	CJM-126 sulfate	C_13_H_10_N_2_O_3_S_2_	307.0211	9.1	Not acquired	R
**Hydroxylation + glucuronidation of M1**						
M11	OH-CJM-126 glucuronide	C_19_H_18_N_2_O_7_S	419.0908	7.4	118.0529, 125.0058, 243.0586	H, M, R
M12	OH-CJM-126 glucuronide	C_19_H_18_N_2_O_7_S	419.0908	7.9	65.0392, 109.0109, 134.0475, 243.0585	H, M, R, D, Mk
M13	OH-CJM-126 glucuronide	C_19_H_18_N_2_O_7_S	419.0908	8.3	65.0392, 109.0109, 134.0475, 243.0585	H, D, Mk
**Hydroxylation + sulfation on M1**						
M14	OH-CJM-126 sulfate	C_13_H_10_N_2_O_4_S_2_	323.0155	7.0	Not acquired	M, R, D
M15	OH-CJM-126 sulfate	C_13_H_10_N_2_O_4_S_2_	323.0155	7.3	118.0528, 125.0058, 243.0588	H, M, R, D
M16	OH-CJM-126 sulfate	C_13_H_10_N_2_O_4_S_2_	323.0155	7.8	Not acquired	H, R, Mk
M17	OH-CJM-126 sulfate	C_13_H_10_N_2_O_4_S_2_	323.0155	9.7	109.0109, 134.0476, 243.0587	H, M, R, D, Mk
**Hydroxylation + *N*-acetylation of M1**						
M18	*N*-Acetyl-OH-CJM-126	C_15_H_12_N_2_O_2_S	285.0692	8.0	81.0341, 118.0527, 125.0056, 243.0587	M, Mk
M19	*N*-Acetyl-OH-CJM-126	C_15_H_12_N_2_O_2_S	285.0692	8.3	81.0341, 118.0527, 125.0056, 243.0587	H, D, Mk
M20	*N*-Acetyl-OH-CJM-126	C_15_H_12_N_2_O_2_S	285.0692	9.3	109.0110, 227.0637	H, R, D, Mk
M21	*N*-Acetyl-OH-CJM-126	C_15_H_12_N_2_O_2_S	285.0692	9.8	109.0109, 243.0589	H, Mk
**Hydroxylation + *N*-acetylation + glucuronidation of M1**						
M22	*N*-Acetyl-OH-CJM-126 glucuronide	C_21_H_20_N_2_O_8_S	461.1013	6.4	Not acquired	Mk
**Dihydroxylation of M1**						
M23	di-OH-CJM-126	C_13_H_10_N_2_O_2_S	259.0541	7.2	Not acquired	H, R
M24	di-OH-CJM-126	C_13_H_10_N_2_O_2_S	259.0541	8.4	Not acquired	H, D
M25	di-OH-CJM-126	C_13_H_10_N_2_O_2_S	259.0541	9.5	Not acquired	Mk
M26	di-OH-CJM-126	C_13_H_10_N_2_O_2_S	259.0541	10.4	Not acquired	D
**Dihydroxylation + sulfation on M1**						
M27	di-OH-CJM-126 sulfate	C_13_H_10_N_2_O_5_S_2_	339.0104	7.0	Not acquired	D
M28	di-OH-CJM-126 sulfate	C_13_H_10_N_2_O_5_S_2_	339.0104	7.2	Not acquired	H, R
M29	di-OH-CJM-126 sulfate	C_13_H_10_N_2_O_5_S_2_	339.0104	8.4	Not acquired	D
**Hydroxylation**						
M30	OH-ETN101	C_16_H_16_N_4_O_2_S	329.1072	1.1	118.0529, 125.0057, 243.0586	H, D, Mk
M31	OH-ETN101	C_16_H_16_N_4_O_2_S	329.1072	1.8	118.0529, 125.0057, 243.0586	H, Mk
M32	OH-ETN101	C_16_H_16_N_4_O_2_S	329.1072	5.5	Not acquired	H, Mk
M33	OH-ETN101	C_16_H_16_N_4_O_2_S	329.1072	7.1	Not acquired	D, Mk
M34	OH-ETN101	C_16_H_16_N_4_O_2_S	329.1072	7.9	Not acquired	Mk

## Data Availability

Data are contained within the article and Supplementary Materials.

## Supplementary Materials

The following supporting information can be downloaded at: https://www.mdpi.com/article/10.3390/metabo14080425/s1, Figure S1: Extracted ion chromatograms of ETN101 and its metabolites identified in mouse hepatocytes incubates; Figure S2: Extracted ion chromatograms of ETN101 and its metabolites identified in rat hepatocytes incubates; Figure S3: Extracted ion chromatograms of ETN101 and its metabolites identified in dog hepatocytes incubates; Figure S4: Extracted ion chromatograms of ETN101 and its metabolites identified in monkey hepatocytes incubates. Table S1: Summary of peak areas for ETN101 and its metabolites from analysis of hepatocyte 1 h incubates (H: human. M: mouse, R: rat, D: dog, Mk: monkey).
