# Supplementary material for: The Comparative Metabolism of a Novel Hepatocellular Carcinoma Therapeutic Agent, 2,3-Diamino-N-(4-(benzo[d]thiazol-2-yl)phenyl)propanamide, in Human and Animal Hepatocytes
Source: Metabolites. 2024 Aug 1;14(8):425. doi: 10.3390/metabo14080425 (PMC11356571; doi:10.3390/metabo14080425)
Supplement: Supplementary file 1 [file metabolites-14-00425-s001.zip › metabolites-3113527-supplementary.pdf]

metabolites

Supplementary Materials

The Comparative Metabolism of a Novel Hepatocellular Carcinoma Therapeutic Agent, 2,3-Diamino-*N*-(4-(benzo[d]thiazol-2-yl)phenyl) propanamide, in Human and Animal Hepatocytes

Young-Heun Jung [1], Dong-Cheol Lee [1], Ye-Min Kwon [1], Eunbee Jang [2], Garam Choi [2], Yeoun-Hee Kim [2], Tae Hwan Kim [3,*] and Ju-Hyun Kim [1,*]

[1] College of Pharmacy, Yeungnam University, Gyeongsan 38541, Republic of Korea; jyoungheun@yu.ac.kr (Y.-H.J.); tbc99156@ynu.ac.kr (D.-C.L.); ym980822@yu.ac.kr (Y.-M.K.)
[2] Etnova Therapeutics, Suwon 16648, Republic of Korea; wkddmsql96@etnova.co.kr (E.J.); garam1458@etnova.co.kr (G.C.); yhkim@etnova.co.kr (Y.-H.K.)
[3] College of Pharmacy, Daegu Catholic University, Gyeongsan 38430, Republic of Korea
* Correspondence: thkim@cu.ac.kr (T.H.K.); jhkim@yu.ac.kr (J.-H.K.)

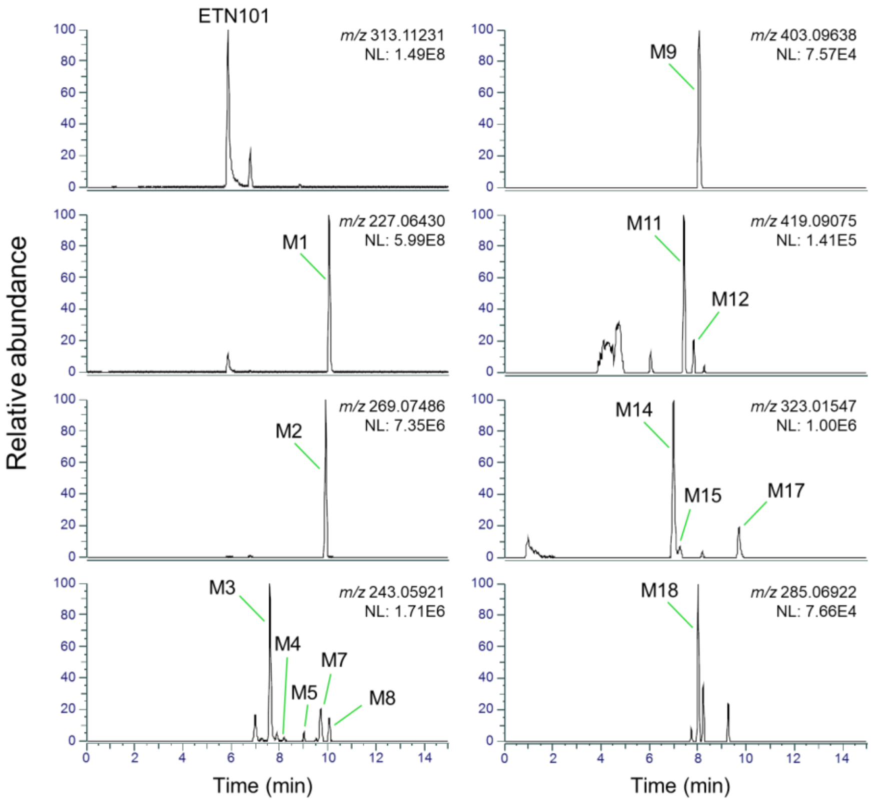

Figure S1. Extracted ion chromatograms of ETN101 and its metabolites identified in mouse hepatocytes incubates

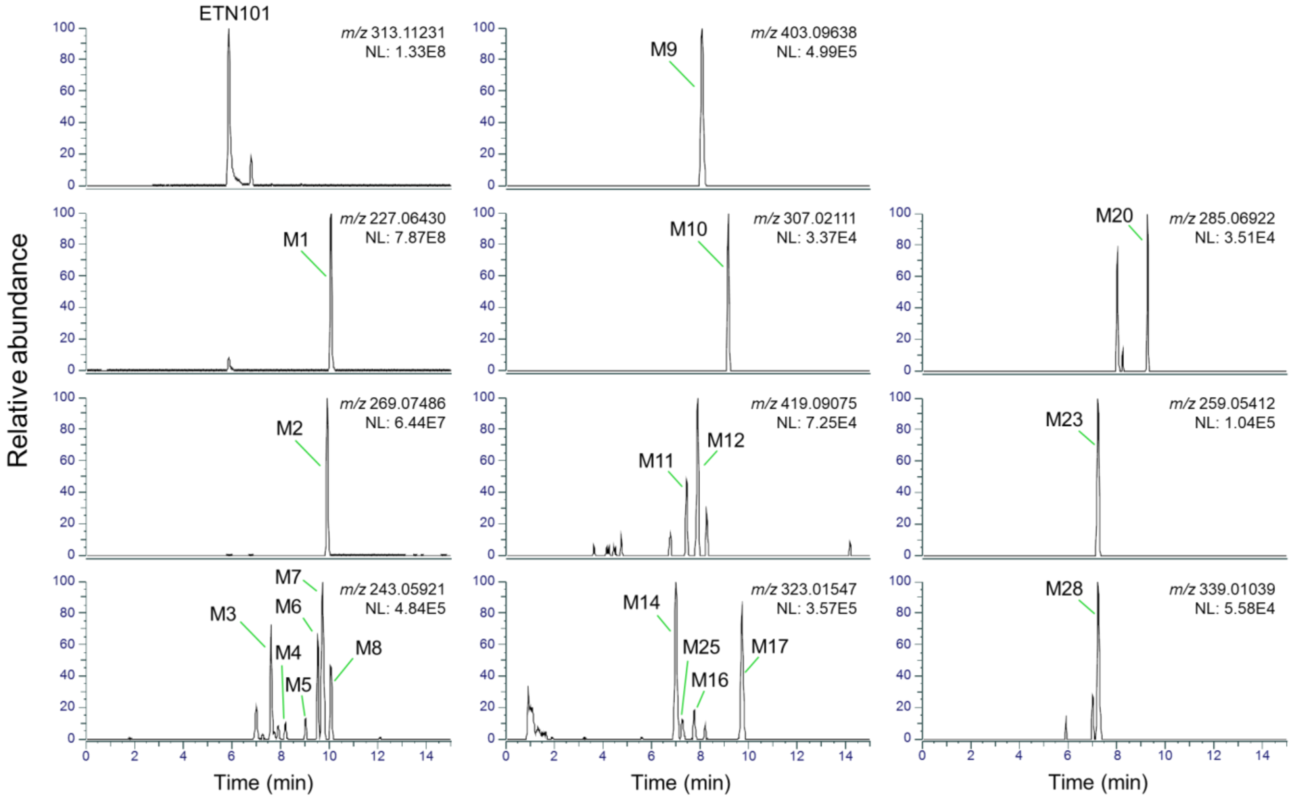

Figure S2. Extracted ion chromatograms of ETN101 and its metabolites identified in rat hepatocytes incubates

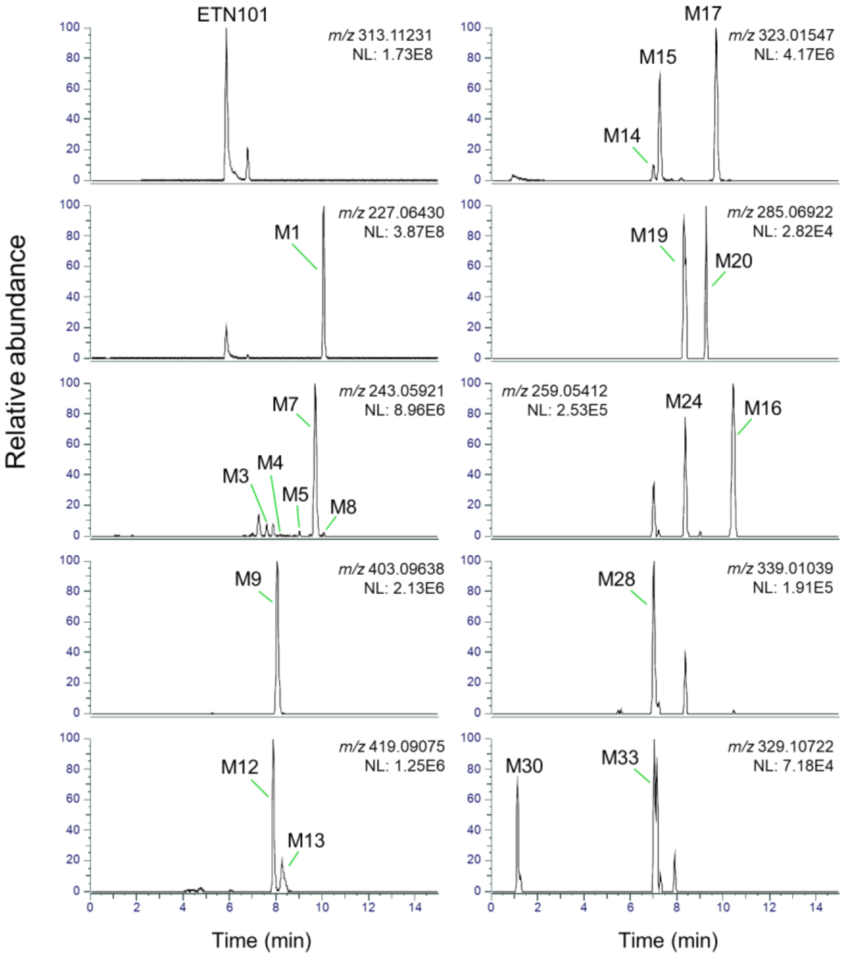

Figure S3. Extracted ion chromatograms of ETN101 and its metabolites identified in dog hepatocytes incubates

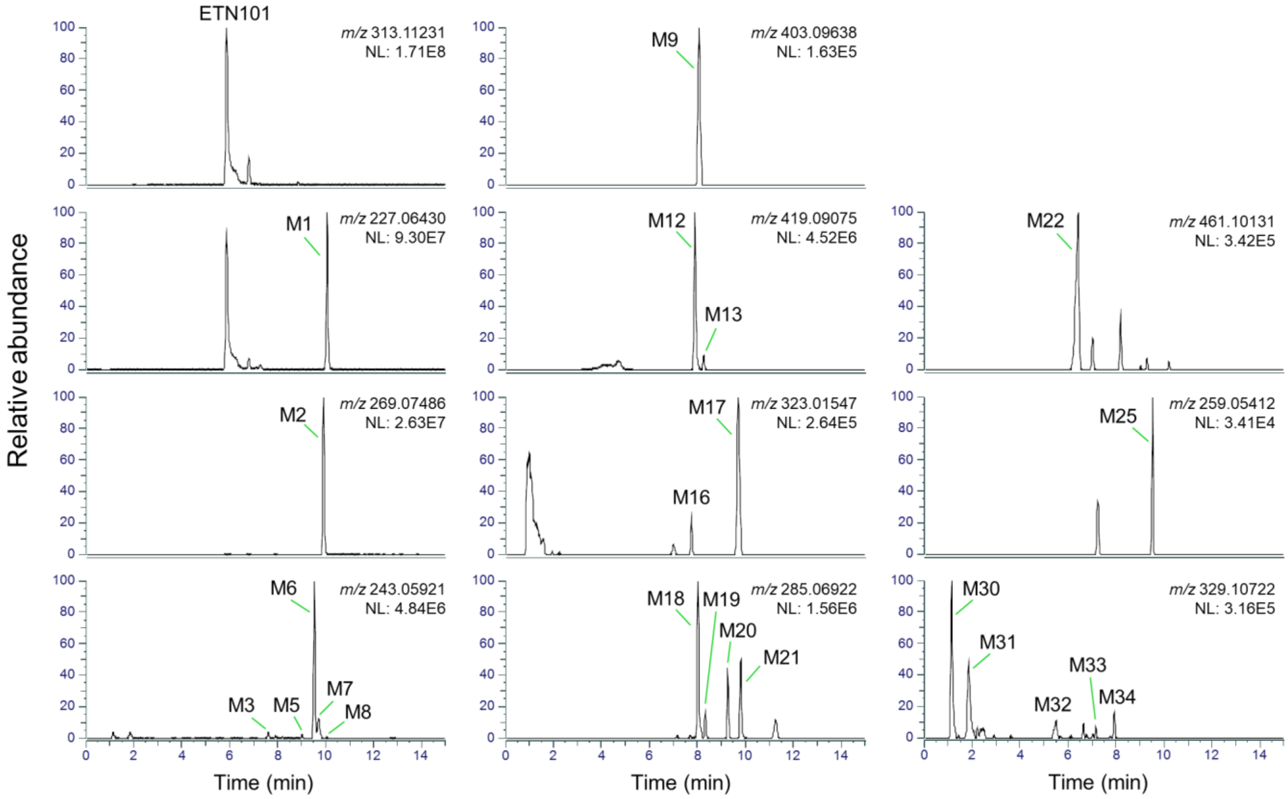

Figure S4. Extracted ion chromatograms of ETN101 and its metabolites identified in monkey hepatocytes incubates

Table S1. Summary of peak areas for ETN101 and its metabolites from analysis of hepatocyte 1 h incubates (H: human. M: mouse, R: rat, D: dog, Mk: monkey).

ID	ETN101 and Metabolites	Elemental composition	Exact mass ($[M+H]^+$)	t_R (min)	Relative peak area (%)				
					Human	Mouse	Rat	Dog	Monkey
	ETN101	$C_{16}H_{16}N_4OS$	313.1123	5.9	76.4	26.8	18.7	39.5	69.0
**Hydrolysis**									
M1	CJM-126	$C_{13}H_{10}N_2S$	227.0643	10.1	21.0	71.8	75.2	55.9	21.5
***N*-acetylation of M1**									
M2	*N*-Acetyl-CJM-126	$C_{15}H_{12}N_2OS$	269.0749	9.9	0.3	0.9	5.7	ND	5.8
**Hydroxylation of M1**									
M3	6-OH-CJM-126	$C_{13}H_{10}N_2OS$	243.0592	7.6	0.1	0.2	< 0.1	0.1	0.1
M4	OH-CJM-126	$C_{13}H_{10}N_2OS$	243.0592	8.2	< 0.1	< 0.1	< 0.1	< 0.1	ND
M5	3′-OH-CJM-126	$C_{13}H_{10}N_2OS$	243.0592	9	0.1	< 0.1	< 0.1	< 0.1	< 0.1
M6	OH-CJM-126	$C_{13}H_{10}N_2OS$	243.0592	9.5	ND	ND	< 0.1	ND	1.1
M7	OH-CJM-126	$C_{13}H_{10}N_2OS$	243.0592	9.7	1.0	0.1	0.1	1.9	0.2
M8	OH-CJM-126	$C_{13}H_{10}N_2OS$	243.0592	10.1	ND	< 0.1	< 0.1	< 0.1	< 0.1
**Glucuronidation of M1**									
M9	CJM-126 glucuronide	$C_{19}H_{18}N_2O_6S$	403.0964	8.1	0.1	< 0.1	0.1	0.5	< 0.1
**Sulfation of M1**									
M10	CJM-126 sulfate	$C_{13}H_{10}N_2O_3S_2$	307.0211	9.1	ND	ND	< 0.1	ND	ND
**Hydroxylation + glucuronidation of M1**									
M11	OH-CJM-126 glucuronide	$C_{19}H_{18}N_2O_7S$	419.0908	7.4	< 0.1	< 0.1	< 0.1	ND	ND
M12	OH-CJM-126 glucuronide	$C_{19}H_{18}N_2O_7S$	419.0908	7.9	0.2	< 0.1	< 0.1	0.2	1.1
M13	OH-CJM-126 glucuronide	$C_{19}H_{18}N_2O_7S$	419.0908	8.3	0.1	ND	ND	0.1	0.1
**Hydroxylation + sulfation on M1**									
M14	OH-CJM-126 sulfate	$C_{13}H_{10}N_2O_4S_2$	323.0155	7	ND	0.2	< 0.1	0.1	ND
M15	OH-CJM-126 sulfate	$C_{13}H_{10}N_2O_4S_2$	323.0155	7.3	< 0.1	< 0.1	< 0.1	0.6	ND
M16	OH-CJM-126 sulfate	$C_{13}H_{10}N_2O_4S_2$	323.0155	7.8	< 0.1	ND	< 0.1	ND	< 0.1
M17	OH-CJM-126 sulfate	$C_{13}H_{10}N_2O_4S_2$	323.0155	9.7	0.5	< 0.1	< 0.1	0.9	0.1
**Hydroxylation + *N*-acetylation of M1**									

M18	*N*-Acetyl-OH-CJM-126	$C_{15}H_{12}N_2O_2S$	285.0692	8	ND	< 0.1	ND	ND	0.4
M19	*N*-Acetyl-OH-CJM-126	$C_{15}H_{12}N_2O_2S$	285.0692	8.3	< 0.1	ND	ND	< 0.1	< 0.1
M20	*N*-Acetyl-OH-CJM-126	$C_{15}H_{12}N_2O_2S$	285.0692	9.3	< 0.1	ND	< 0.1	< 0.1	0.1
M21	*N*-Acetyl-OH-CJM-126	$C_{15}H_{12}N_2O_2S$	285.0692	9.8	< 0.1	ND	ND	ND	0.2
**Hydroxylation + *N*-acetylation + glucuronidation of M1**									
M22	*N*-Acetyl-OH-CJM-126 glucuronide	$C_{21}H_{20}N_2O_8S$	461.1013	6.4	ND	ND	ND	ND	0.2
**Dihydroxylation of M1**									
M23	di-OH-CJM-126	$C_{13}H_{10}N_2O_2S$	259.0541	7.2	< 0.1	ND	< 0.1	ND	ND
M24	di-OH-CJM-126	$C_{13}H_{10}N_2O_2S$	259.0541	8.4	< 0.1	ND	ND	< 0.1	ND
M25	di-OH-CJM-126	$C_{13}H_{10}N_2O_2S$	259.0541	9.5	ND	ND	ND	ND	< 0.1
M26	di-OH-CJM-126	$C_{13}H_{10}N_2O_2S$	259.0541	10.4	ND	ND	ND	0.1	ND
**Dihydroxylation + sulfation on M1**									
M27	di-OH-CJM-126 sulfate	$C_{13}H_{10}N_2O_5S_2$	339.0104	7	ND	ND	ND	< 0.1	ND
M28	di-OH-CJM-126 sulfate	$C_{13}H_{10}N_2O_5S_2$	339.0104	7.2	< 0.1	ND	< 0.1	ND	ND
M29	di-OH-CJM-126 sulfate	$C_{13}H_{10}N_2O_5S_2$	339.0104	8.4	ND	ND	ND	< 0.1	ND
**Hydroxylation**									
M30	OH-ETN101	$C_{16}H_{16}N_4O_2S$	329.1072	1.1	< 0.1	ND	ND	< 0.1	0.1
M31	OH-ETN101	$C_{16}H_{16}N_4O_2S$	329.1072	1.8	0.1	ND	ND	ND	0.1
M32	OH-ETN101	$C_{16}H_{16}N_4O_2S$	329.1072	5.5	< 0.1	ND	ND	ND	< 0.1
M33	OH-ETN101	$C_{16}H_{16}N_4O_2S$	329.1072	7.1	ND	ND	ND	< 0.1	< 0.1
M34	OH-ETN101	$C_{16}H_{16}N_4O_2S$	329.1072	7.9	ND	ND	ND	ND	< 0.1

ND:<1.2E+05

Relative peak area (%) = (peak area of identified metabolite / sum of peak areas of all identified metabolites in the species) × 100

Relative peak area values may vary with the identification of additional metabolites.
